# A Method to Improve the Distribution of Observations in GNSS Water Vapor Tomography

**DOI:** 10.3390/s18082526

**Published:** 2018-08-02

**Authors:** Fei Yang, Jiming Guo, Junbo Shi, Lv Zhou, Yi Xu, Ming Chen

**Affiliations:** 1 School of Geodesy and Geomatics, Wuhan University, Wuhan 430079, China; coffeeyang@whu.edu.cn (F.Y.); jbshi@sgg.whu.edu.cn (J.S.); zhoulv@whu.edu.cn (L.Z.); dhxzyz@whu.edu.cn (Y.X.); cm@ngcc.cn (M.C.); 2 Key Laboratory of Precise Engineering and Industry Surveying of National Administration of Surveying, Mapping and Geoinformation, Wuhan University, Wuhan 430079, China; 3 Research Center for High Accuracy Location Awareness, Wuhan University, Wuhan 430079, China; 4 College of Geomatics and Geoinformation, Guilin University of Technology, Guilin 541004, China; 5 National Geomatics Center of China, Beijing 100830, China

**Keywords:** GNSS remote sensing, atmospheric sounding, water vapor tomography, meteorology

## Abstract

Water vapor is an important driving factor in the related weather processes in the troposphere, and its temporal-spatial distribution and change are crucial to the formation of cloud and rainfall. Global Navigation Satellite System (GNSS) water vapor tomography, which can reconstruct the water vapor distribution using GNSS observation data, plays an increasingly important role in GNSS meteorology. In this paper, a method to improve the distribution of observations in GNSS water vapor tomography is proposed to overcome the problem of the relatively concentrated distribution of observations, enable satellite signal rays to penetrate more tomographic voxels, and improve the issue of overabundance of zero elements in a tomographic matrix. Numerical results indicate that the accuracy of the water vapor tomography is improved by the proposed method when the slant water vapor calculated by GAMIT is used as a reference. Comparative results of precipitable water vapor (PWV) and water vapor density (WVD) profiles from radiosonde station data indicate that the proposed method is superior to the conventional method in terms of the mean absolute error (MAE), standard deviations (STD), and root-mean-square error (RMS). Further discussion shows that the ill-condition of tomographic equation and the richness of data in the tomographic model need to be discussed separately.

## 1. Introduction

Water vapor accounts for a small percentage of the troposphere, but it is the most active meteorological element, and significantly impacts the atmosphere and plays a key role in various climates and a series of weather phenomena. Measuring and monitoring the water vapor contents and movements, and understanding the temporal-spatial change in water vapor, are important for meteorological research and applications such as precipitation forecasts and extreme weather warnings.

The development of Global Navigation Satellite System (GNSS) reference station networks provides rich data sources of high spatiotemporal resolution to monitor water vapor. Bevis et al. [1] initially proposed the remote sensing of atmospheric water vapor using GNSS. To achieve spatiotemporal resolution of water vapor information in the research region, a tomographic technique has been widely applied in GNSS meteorology. This method is developed from the medical field [2] in which the density distribution of an object is obtained by X-ray projections. It has been applied to the research of geology [3], earthquakes [4], gas tracing [5], and ionosphere modeling [6]. Since the application of troposphere tomography in 4-D wet refractive index first realized by Flores et al. [7], the development of GNSS water vapor tomography has advanced significantly. Hirahara et al. [8] used a damped least squares method to reveal the spatial and temporal change of refractivity. Nilsson et al. [9] investigated the impact of tomographic factors such as the number of GNSS stations, elevation cutoff angle, and type of GNSS through simulations. Song et al. [10] introduced the posterior variance component estimation method to determine the weights of the tomographic equation. Rohm and Bosy [11] used the Moore-Penrose pseudo-inverse of a variance-covariance matrix in a local tomography troposphere model over a mountainous area. Bender [12] proposed an algebraic reconstruction technique for GNSS tomographic modeling. Adavi et al. [13] used a hybrid regularization technique to reconstruct profiles of water vapor without constraint equations. Ding et al. [14] proposed an innovative node parameterization approach in water vapor tomography using a combination of three meshing techniques.

The distribution of observations in the above mentioned tomographic approaches is relatively concentrated due to the special structures of GNSS satellite constellations and the unfavorable geometry of GNSS stations in the tomographic region, as well as the specific selection of tomography period (usually 30 min). Unfortunately, some voxels cannot be penetrated by sufficient signal rays [15,16,17]. To overcome the ill-posed issue, Xia et al. [18] exploited Constellation Observing System for Meteorology, Ionosphere, and Climate (COSMIC) occultation data for water vapor tomography. Benevides et al. [19] introduced the water vapor information of Interferometric Synthetic Aperture Radar (InSAR) into GNSS troposphere tomography. Jiang et al. [20] improved the distribution of observations by assimilating the measurements of surface water vapor into GNSS tomography. Chen and Liu [21] assessed the performance of troposphere tomography using multi-source water vapor information, such as radiosonde, GPS, water vapor radiometer (WVR), and numerical weather predictions. Although the combination of various water vapor observation technologies can provide additional observation information, they are still hard to carry out in practice when the cost, spatiotemporal resolution, and other restrictions are considered. COSMIC occultation events occur only at specific times, and the return period of SAR satellites reaches several days, resulting in low temporal resolution of these techniques. The measurements of surface water vapor are unavailable in most tomographic experiments, given that many GNSS receiver stations are not equipped with meteorological equipment. The numerical weather prediction system, such as weather research and forecasting (WRF) models, provide meteorological products with only a spatial resolution of 3 km × 3 km and a time resolution of 1 h. The time resolution and accuracy of the atmospheric water vapor information obtained by WVR are extremely high, but they are difficult to maintain due to high equipment costs. Therefore, a method to improve the distribution of observations by introducing virtual observations is proposed. The virtual observations are acquired using a specific elevation and azimuth angle, based on the wet mapping function and the wet delay gradient in the east–west and north–south directions. GNSS water vapor tomography is estimated with a different method, which is validated by slant water vapor and radiosonde data.

This paper is organized as follows. Tomography theory and methods for improving the distribution of observations are described in Section 2. Section 3 presents the tomography experiments and results analysis. The discussion and conclusion are presented in Section 4 and Section 5, respectively.

## 2. Tomography Theory and Method

### 2.1. Water Vapor Tomography Modeling

Zenith total delay (ZTD) is an average parameter in spatial aspects and can be obtained from GNSS observations. The zenith wet delay (ZWD) is the wet component of ZTD affected by water vapor content along the signal travel path and can be retrieved by extracting the zenith hydrostatic delay (ZHD) from ZTD using the formula ZWD = ZTD − ZHD. The empirical model used to calculate the corresponding ZHD is as follows [22]:

(1) $$\mathrm{ZHD}=\frac{0.002277\times P_{s}}{1-0.00266\times \cos\left(2\varphi \right)-0.00028\times H}$$

where $P_{s}$ (unit: hPa) is the surface pressure, which can be obtained from measured meteorological data or calculated by the Global Pressure and Temperature Model (GPT). φ and H (unit: km) denote the latitude and the geodetic height of the station, respectively.

ZWD can be translated into PWV on the basis of a linear relationship by using the following formula [23]:

(2) $$\mathrm{PWV}=\Pi \times \mathrm{ZWD}=\frac{10^{6}}{\rho_{w}\times \frac{R}{m_{w}}\left(\frac{k_{3}}{T_{m}}+k_{2}-\frac{m_{w}}{m_{d}}\times k_{1}\right)}\times \mathrm{ZWD}$$

where Π is the conversion factor. $\rho_{w}$ represents the liquid water density (unit:$g\cdot m^{-3}$); R indicates the universal gas constant ($R=8314\;\mathrm{Pa}\cdot m^{3}\cdot K^{-1}\cdot \mathrm{kmo}l^{-1}$); $m_{w}$ and $m_{d}$ are the molar mass of water and the dry atmosphere ($m_{w}=18.02\;\mathrm{kg}\cdot \mathrm{kmo}l^{-1}$, $m_{d}=28.96\;\mathrm{kg}\cdot \mathrm{kmo}l^{-1}$), respectively. $k_{1}$, $k_{2}$, and $k_{3}$ are empirical physical constants ($k_{1}=77.604\;K\cdot \mathrm{hP}a^{-1}$, $k_{2}=70.4\;K\cdot \mathrm{hP}a^{-1}$, $k_{3}=3.775\times 10^{5}\;K^{2}\cdot \mathrm{hP}a^{-1}$); and $T_{m}$ (unit: K) denotes the weighted mean temperature, which is a function of vapor pressure and temperature at different altitude. In practice, it can be approximated using a measurement of the surface temperature $T_{s}$ in K ($T_{m}=85.63+0.668T_{s}$) [24,25].

The slant water vapor is the integrated water vapor along the slant path between the ground receiver and GNSS satellite; it is expressed as follows:

(3) $$\mathrm{SWV}=\int_{s}\rho_{v}ds=\mathrm{PWV}\times f\left(ele\right)+\Pi \times f\left(ele\right)\times \cot\left(ele\right)\times \left(G_{NS}^{w}\times \cos\left(azi\right)+G_{WE}^{w}\times \sin\left(azi\right)\right)+R$$

where s is the path of a satellite signal ray, $\rho_{v}$ represents the water vapor density (unit: $g\cdot m^{-3}$), f is the wet mapping coefficient. ele and azi are the satellite elevation and azimuth, respectively. $G_{NS}^{w}$ and $G_{WE}^{w}$ are the wet delay gradient parameters in the north-east and east-west directions, respectively. R refers to the non-homogeneous variation of water vapor, it is calculated by multiplying the post-fit residual by the conversion factor [26,27].

**Figure 1:**
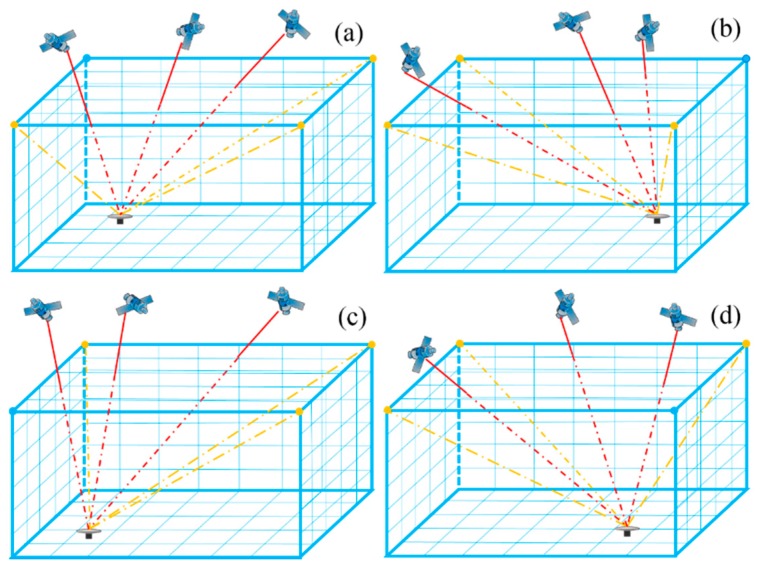
3-D distribution of slant water vapor and virtual slant water vapor. (**a**–**d**) represent the GNSS receiver located in four different tomographic regions, namely northwest, northeast, southwest, and southeast.

The observation equation, which is based on the unknown water vapor density within each tomographic voxel and the distances of GNSS signal rays crossing the divided voxel (red paths shown in Figure 1), is established as follows:

(4) $$\mathrm{SWV}=\sum_{i=1}^{n}d_{i}\times x_{i}$$

where SWV denotes the slant water vapor that can be acquired by the tropospheric estimation. $d_{i}$ denotes the distance of signal rays inside voxel i, which can be achieved by the station and satellite coordinates, and $x_{i}$ is the unknown parameter, which denotes water vapor density of voxel i.

A spatial relation exits between water vapor in a specific voxel and its surrounding ones. In the horizontal direction, Rius et al. [28] pointed out that the distribution of water vapor density is relatively stable within a small region. Thus, water vapor density within a voxel is related to the weighted average of its adjacent voxels in the same grid layer. The horizontal constraint equation, which stabilizes the tomographic result, is constructed by the Gaussian inverse distance weighted function [29]:

(5) $$w_{1}x_{1}+w_{2}x_{2}+\cdots +w_{i-1}x_{i-1}-x_{i}+w_{i+1}x_{i+1}+\cdots w_{m}x_{m}=0$$

where $w_{i-1}=d_{i-1,i}/\sum_{j=1}^{m}d_{i-1,j}$ is the horizontal weighted coefficient, $d_{i-1,i}$ represents the distance between voxel i−1 and i, and m denotes the total number of voxels in the same layer.

To reasonably describe the vertical profile information of the water vapor, vertical constraints must be added. Otherwise, the tomographic results cannot be distinguished before and after the exchange of two arbitration layers of the tomography grid. The analysis of meteorological data for many years reveals that the vertical constraint equation is established by the form of exponential function [30,31]:

(6) $$x_{j}-e^{\left(h_{j+m}-h_{j}\right)/H}\times x_{j+m}=0$$

where $x_{j}$ and $h_{j}$ denote the water vapor density and corresponding height within voxel j, respectively. $x_{j+m}$ and $h_{j+m}$ represent that of voxel j+m. H is the water vapor scale height, which ranges from 1 to 2 km.

### 2.2. Method for Improving Distribution of Observations

Only the GNSS signal rays passing through from the top boundary of the tomographic region can be used to construct observation equations. Thus, most of the rays in the low elevation angle, which contains abound water vapor information in low layer voxels and boundary voxels, are eliminated. In addition, most of the signal rays used to construct the observation equation only penetrate the vertical voxels above the GNSS stations. Therefore, the distribution of observations in the tomography equations is relatively centralized, resulting in many voxels without signal rays passing through and in overabundance of zero elements in the tomographic matrix. In this paper, virtual observations in specific elevation and azimuth angle (yellow paths shown in Figure 1) are introduced to improve the distribution of observations. Boundary points are selected, coordinates are transformed, and the wet mapping function and the wet delay gradient in the north–south and east–west directions are used in this method. The corresponding procedure is as follows:

1. The direction of the GNSS station in the tomographic area (northwest, southwest, southeast, and northeast) is determined.
2. The GNSS stations are selected as the starting points by considering the boundary points at the top of the tomographic region as the terminal points of the virtual observations.
3. The geodetic coordinate is converted to a site-centric coordinate.
  (7) BLH→NEU 
4. The elevation $ele^{\prime }$ and azimuth $azi^{\prime }$ of the virtual observations are calculated as follows:
  (8) $$ele^{\prime }=\arctan\left(\frac{U_{t}}{\sqrt{{N_{t}}^{2}+{E_{t}}^{2}}}\right)$$
  (9) $$azi^{\prime }=\arctan\left(\frac{E_{t}}{N_{t}}\right)$$
  where $N_{t},E_{t},U_{t}$ represent the coordinate of the terminal point in the north, east, and zenith directions, respectively, of the site-centric coordinate, which can be obtained from the information of tomography boundary.
5. The virtual slant water vapor $\mathrm{SW}V^{\prime }$ is calculated, as follows:
  (10) $$\mathrm{SW}V^{\prime }=f\left(ele^{\prime }\right)\times \mathrm{PWV}+\Pi \times f\left(ele^{\prime }\right)\times \cot\left(ele^{\prime }\right)\times \left(G_{NS}^{w}\times \cos\left(azi^{\prime }\right)+G_{WE}^{w}\times \sin\left(azi^{\prime }\right)\right)$$
6. The virtual observation equation is constructed to improve the distribution of observations, as follows:
  (11) $$\mathrm{SW}V^{\prime }=\sum_{i=1}^{n}d_{i}^{\prime }\times x_{i}$$
  where $d_{i}^{\prime }$ is the distance of the virtual slant water vapor inside voxel i, which can be calculated using these coordinates.
7. The new functional model of water vapor tomography is constructed and the water vapor density in each voxel is solved using the least square method:
  (12) $$\left[\begin{array}{c} y_{swv}\\ y_{h}\\ y_{v}\\ y_{swv^{\prime }} \end{array}\right]=\left[\begin{array}{c} A_{swv}\\ A_{h}\\ A_{v}\\ A_{swv^{\prime }} \end{array}\right]\times X+\left[\begin{array}{c} \Delta_{swv}\\ \Delta_{h}\\ \Delta_{v}\\ \Delta_{swv^{\prime }} \end{array}\right]$$
  where $y_{swv},y_{swv^{\prime }}$ are the column vectors with a set of SWV and virtual SWV, respectively; *y_h_*, *y_v_* are the column vectors with the value of 0 for horizontal and vertical constraint equations, respectively; *A_SWV_*, *A_h_*, *A_v_*, *A_SWV’_* represent the coefficient matrices of observation equation, horizontal constraint equation, vertical constraint equation, and virtual observation equation, respectively;$X={\left[x_{1},x_{2}\cdots x_{n}\right]}^{T}$ is a vector of water vapor to be estimated; and $\Delta_{swv},\Delta_{h},\Delta_{v},\Delta_{swv^{\prime }}$ denote the noises of observation equation, horizontal constraint equation, vertical constraint equation, and virtual observation equation, respectively.

## 3. Experiment and Analysis

### 3.1. Experiment Description and GNSS Data Processing Strategy

**Figure 2:**
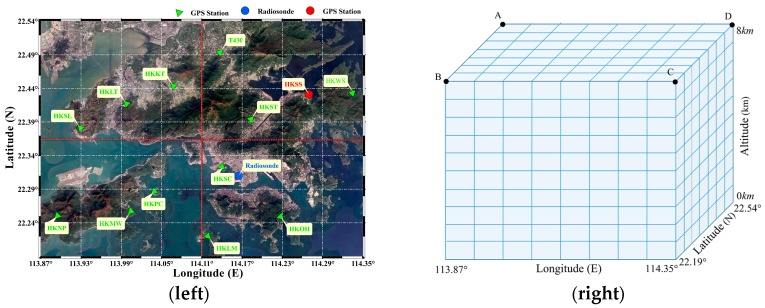
Geographic distribution of GNSS, radiosonde station (**left**) and 3-D tomographic voxels (**right**).

As shown in Figure 2, Hong Kong was selected as the tomography area, the scope of which was 113.87°–114.35° for longitude, 22.19°–22.54° for latitude and 0–8.0 km for altitude. The vertical resolution was 800 m and the horizontal resolution was 0.06° in longitude, and 0.05° in latitude. A tomography grid containing 8 × 7 × 10 voxels was obtained. To determine which boundary point to use when improving the distribution of observations, the tomography area was divided into four regions (northwest, southwest, southeast, and northeast). Twelve GNSS stations of the Hong Kong Satellite Positioning Reference Station Network (SatRet), which were evenly distributed in the four regions, were selected in the tomography modeling. Another GNSS station and radiosonde station (45004) were used to check the result of the water vapor tomography. The information of each site was shown in Table 1. A, B, C, and D in the table represent boundary points and their geodetic coordinates were (22.54°, 113.87°, 8.0 km), (22.19°, 113.87°, 8.0 km), (22.19°, 114.35°, 8.0 km), and (22.54°, 114.35°, 8.0 km). To validate the proposed method, GNSS observation data of DOY 182 to 188, 2017 were selected with a sampling rate of 30 s. The data from a radiosonde station at 00:00 and 12:00 UTC was collected when the sounding balloon was launched daily. In this paper, two schemes, including the conventional method, which was considered as Scheme #1, and the proposed method which was regarded as Scheme #2, are determined.

The GAMIT 10.61 software was adopted for the GNSS troposphere estimation based on the double-differenced model. Tropospheric parameters among the network were strongly correlated because of the presence of a short baseline between GNSS receivers in the tomographic area. Few International GNSS Service (IGS) stations (BJFS, LHAZ, and SHAO) were incorporated into the solution model to reduce this correlation [32,33,34]. The GAMIT processing strategy for tropospheric estimation is summarized in Table 2. LC_AUTCLN indicates that the observable was the ionosphere-free linear combination, and BASELINE shows that orbital or EOP parameters were not estimated. The unknown parameters, including ZTD at 2-h intervals and troposphere delay gradients at 4-h intervals, are estimated using the least square method. By interpolation, ZTD and troposphere delay gradients with 30 s sampling rate are obtained. To achieve the wet delay gradients, Bar-Sever et al. [35] regarded the average of troposphere gradients within 12 h as the dry delay gradients and subtracted it from troposphere delay gradients. ZTD was converted to PWV using Equations (1) and (2), and SWV and virtual SWV were obtained using Equations (3) and (10), respectively.

### 3.2. Analysis of the Proposed Method

**Figure 3:**
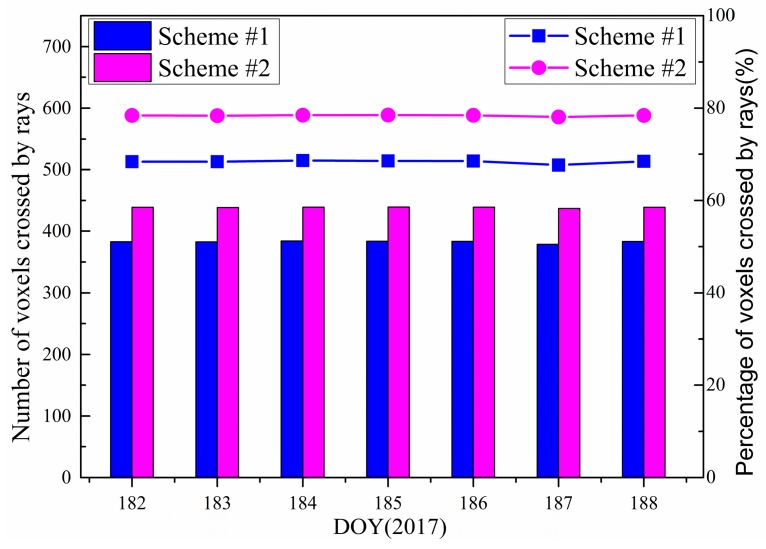
Average number (histogram) and percentage (line) of voxels crossed by signal rays for Scheme #1 and Scheme #2 of DOY 182 to 188, 2017.

**Figure 4:**
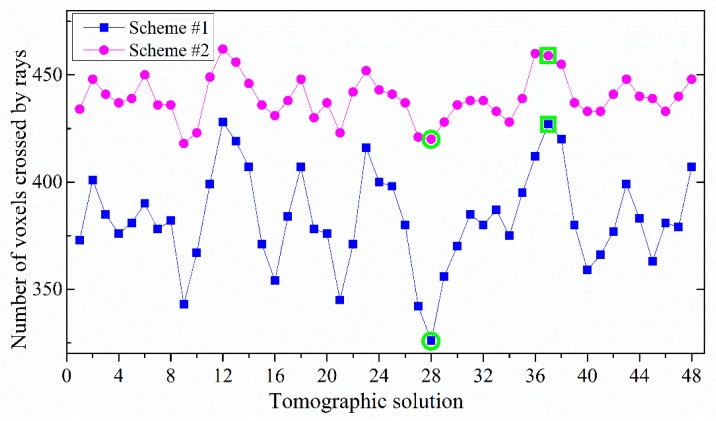
Number of voxels crossed by signal rays in two schemes for each tomographic solution at DOY 182, 2017.

For each tomographic solution, the period covered was 0.5 h, and the sampling rate was 30 s. A total of 3 × 60 × 12 virtual SWVs could be constructed within the 12 GNSS stations. To analyze the influence of the proposed method in Scheme #2 on the number of voxels, where signal rays passed through, relevant experiments were conducted. The corresponding results for Scheme #1 and Scheme #2 are shown in Figure 3. It can be seen that Scheme #2 allowed for more voxels that were passed through by rays. Specifically, the average number of crossed voxels was raised from 382 to 438, and the percentage of total voxels was increased from 68% to 78% in this period. Figure 4 shows the results per 30 min at DOY of 182, 2017. Obviously, the number of voxels that were passed through by signal rays was improved by exploiting the virtual SWV of Scheme #2. The largest increase occurred in the 28th solution, reaching 94 voxels (Solution #a, highlight by green circle), whereas the 37th solution had the least increase, reaching only 32 voxels (Solution #b, highlight by green squares). The average increase was 56 voxels at DOY of 182, 2017.

**Figure 5:**
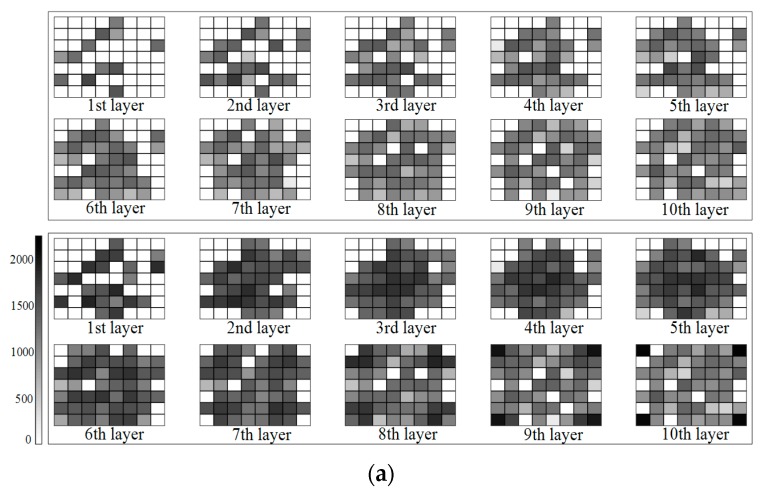

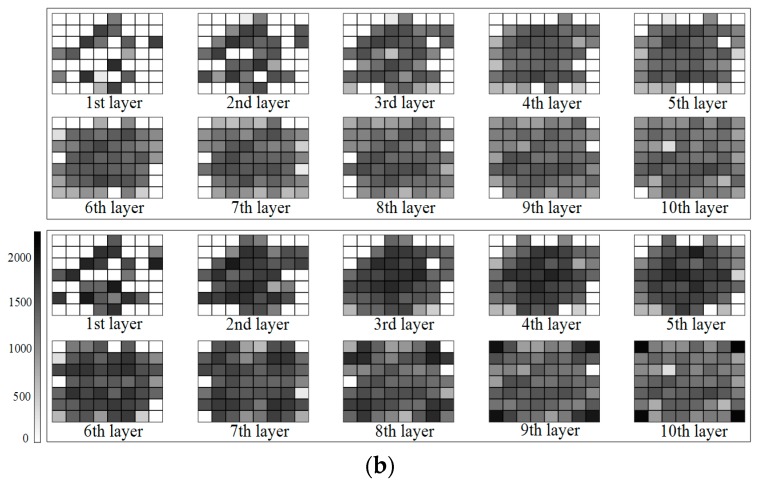
(**a**) Grayscale graph of the number of signal rays passing through each voxel in Solution #a; (**b**) Grayscale graph of the number of signal rays passing through each voxel in Solution #b.

To further illustrate the effects of the proposed method, Figure 5 shows the grayscale graph of the number of signal rays passing through each voxel. In this graph, the deepening of the grayscale represents an increase in the number of rays passing through the voxel (white represents no ray through). Thus, it can also be used to display the specific location of the increased voxel crossed by virtual SWV of Scheme #2. (a) and (b) stand for Solutions #a and #b, respectively. The upper one adopted Scheme #1 and the lower one utilized the Scheme #2 in each graph. It can be seen that the addition of virtual SWV can effectively supplement the situation of no signal rays passing through the voxels around the GNSS stations. In the high level tomographic layer, more voxels in the tomographic boundary region were penetrated by signal rays because of the virtual SWV of Scheme #2, which was a good complement to Scheme #1. However, the change of gray in the images reveals that the number of rays passing through voxels was significantly increased with the addition of virtual SWV. Table 3 lists the detailed values corresponding to Figure 5, and these values were counted from the perspective of the layer.

### 3.3. Comparison with SWV of Station HKSS

To assess the performance of the proposed method, SWV achieved by GNSS observation data and meteorological data was compared with SWV calculated from tomographic results of different schemes. Table 4 shows the average MAE, STD, and RMS derived from the two schemes and the statistical results for 7 days. The results reveal that MAE, STD, and RMS values of Scheme #2 were lower than those of Scheme #1 for every day. The average MAE was decreased by 1.79 mm from 10.53 mm to 8.74 mm, whereas STD and RMS were reduced by 1.65 and 2.45 mm from 11.35/14.09 mm to 9.70/11.64 mm, respectively. This indicates that Scheme #2 had a higher average accuracy than Scheme #1.

**Figure 6:**
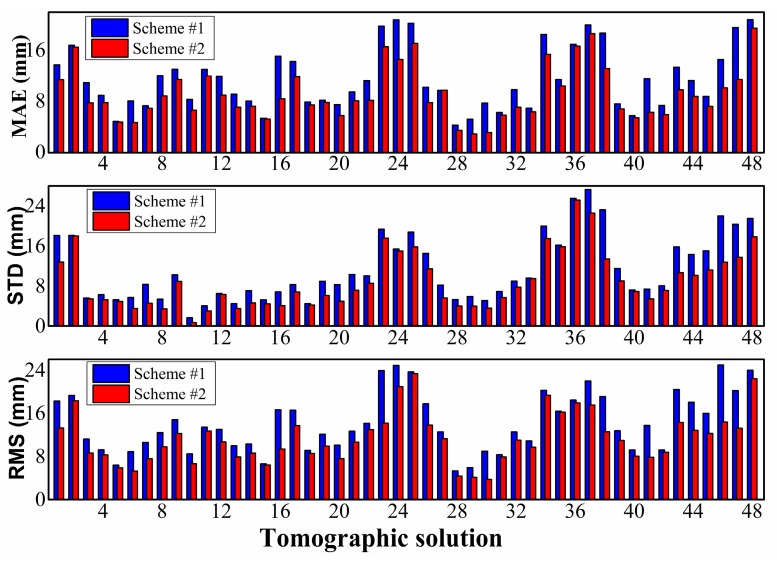
Accuracies in terms of MAE (**top**), STD (**middle**), and RMS (**bottom**) using two schemes in every tomographic solution of DOY 182, 2017.

To specifically reveal the superiority of the proposed method (Scheme #2), the MAE, STD, and RMS of every tomographic solution in DOY 182, 2017 are shown in Figure 6. The evolution is developed from the perspective of each tomographic result of 30 mins. The advantage of the proposed method (Scheme #2) was evident in terms of smaller MAE, STD, and RMS for every tomographic solution of DOY 182, 2017. The maximum and minimum MAE/STD/RMS of Scheme #1 were 20.838/27.271/24.894 and 4.310/1.643/5.353 mm, respectively, and those of Scheme #2 were 19.518/22.599/20.955 and 3.499/0.683/4.119 mm, respectively.

### 3.4. Comparison with Radiosonde Data

**Figure 7:**
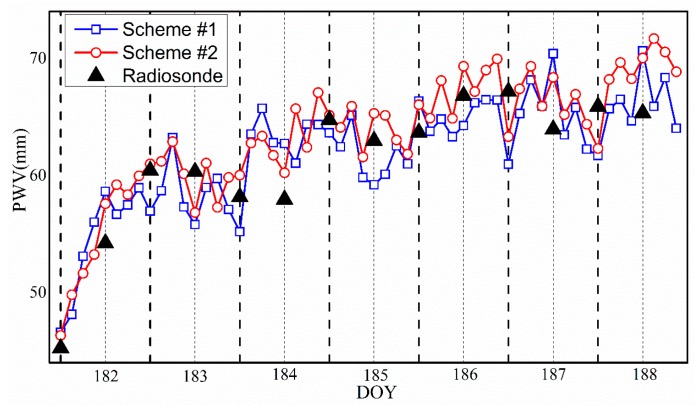
Comparison of PWV time series derived from various tomographic schemes and radiosonde data for the period of DOY 182 to 188, 2017.

The PWV calculated from radiosonde was suitable as a standard for evaluating the accuracy of the tomographic results. Thus, the PWV over the radiosonde station was also computed by the water vapor density of voxels from different tomographic schemes. In this experiment, eight tomographic solutions were selected daily to calculate PWV. Figure 7 presents the PWV time series of Scheme #1 and Scheme #2 and their comparison with radiosonde data. It can be seen that the trend of the PWV time series was basically consistent. Compared with radiosonde data, the two schemes match, and Scheme #2 was more consistent than Scheme #1. The statistical results of PWV differences between radiosonde data and tomography are listed in Table 5. Scheme #2 had an advantage compared with Scheme #1 in terms of MAE, STD, and RMS.

**Figure 8:**
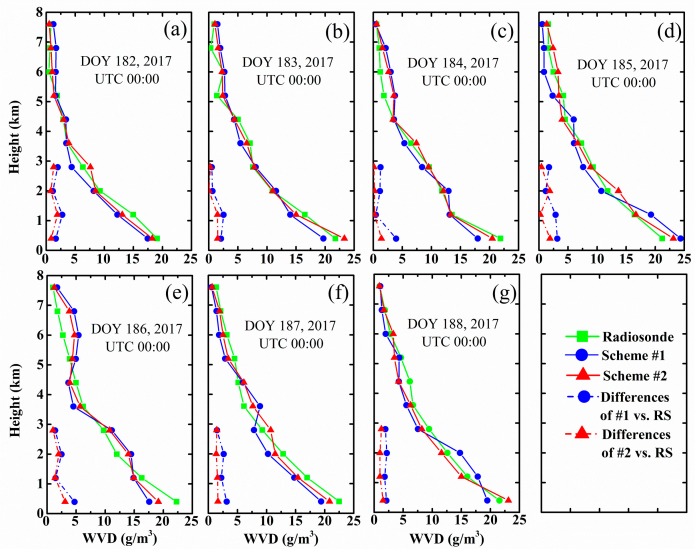
(**a**–**g**) represent water vapor density profile comparisons between radiosonde and different schemes at UTC 00:00 from DOY 182 to 188, 2017.

Although the PWV time series derived from Scheme #2 was in better agreement with the radiosonde data, while low MAE, STD and RSM were shown, we may not conclude that the vertical water vapor distribution of Scheme #2 was better than that of Scheme #1 because the PWV value was unchanged if two vertical layers were exchanged arbitrarily. Thus, the water vapor density profile should be compared between the two schemes and radiosonde data during the tomographic period. The data of UTC 00:00 and 12:00 of DOY 182 to 188, 2017 were selected to further test the accuracy of the tomographic results from different schemes. Figure 8 represents the WVD profiles for different altitudes at individual dates. It can be seen that the WVD obtained by Schemes #1 and #2 were in conformity with that derived from radiosonde. Evidently, the red line was more consistent with the green line than the blue one, thereby suggesting that the WVD of the proposed method better matches the radiosonde. This consistency was particularly evident at the bottom altitudes and implies that the proposed method achieves better water vapor density than the conventional method. Figure 8 shows the differences between the results of Schemes #1 and #2 and the radiosonde data in the low-altitude atmosphere from the red and blue dashed lines. It could be more clearly found that the differences between the result of Scheme #2 and radiosonde data were smaller than that of Scheme #1. Table 6 lists the statistical results of the water vapor density profile comparison between radiosonde and different schemes at UTC 12:00 from DOY 182 to 188, 2017, showing consistency with Figure 8. Thus, tomographic results of Scheme #2 were better than those of Scheme #1.

**Figure 9:**
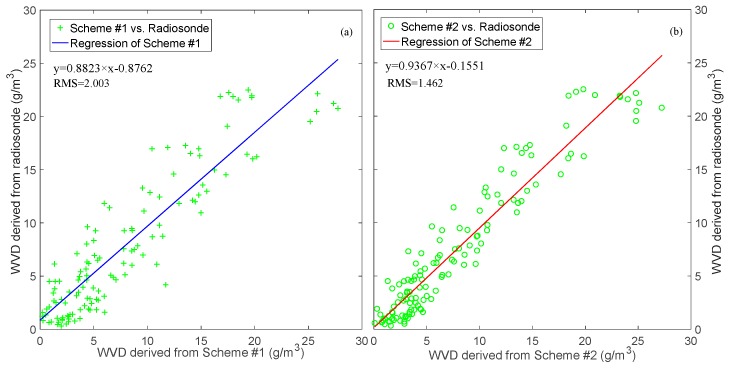
Linear regression of water vapor density from radiosonde and two schemes. (**a**) and (**b**) represent the regression results of Scheme #1 and #2, respectively.

To explore the overall accuracy of WVD obtained by different schemes, all values from radiosonde data in the experimental period were selected for linear regression analysis. Figure 9 represents the linear regression results of the two schemes, in which the scatter points of the right graph were closer to the corresponding straight lines. The linear regression coefficient and RMS of each scheme were achieved, and the regression equations were established. Compared with the result of Scheme #1, the starting point of the regression equation was closer to 0, and the slope was improved in Scheme #2. The numerical results show that the RMS and slope were 2.003 g·m^−3^/0.8823 and 1.462 g·m^−3^/0.9367 in the two schemes, respectively. The regression analysis further indicates that the tomographic accuracy of Scheme #2 was superior to that of Scheme #1.

## 4. Discussion

The proposed method allowed the signal rays to pass through more voxels, thereby enriching the voxels’ information in the tomographic region and improving the distribution of observations. The different tomography results were compared with the radiosonde data and SWV, and it was found that the proposed method could effectively improve the accuracy of results. In order to analyze whether the ill-conditioned nature of the tomography model is weakened when the distribution of the observation is improved, the concept of matrix condition number is introduced. The condition number measures the degree of dispersion of the eigenvalues of the coefficient matrix, which can be used to diagnose the ill-posed problem of matrix. When the condition number of a matrix is high, then the ill-condition becomes serious [36]. It is defined as follows:

(13) $$\kappa \left(N\right)=\left\|N^{-1}\right\|\times \left\|N\right\|$$

where $N=A^{T}A$, κ(N) denotes the condition number of matrix N, and ‖N‖ is the norm of matrix N.

With the addition of various types of equations, the change in the condition number of the coefficient matrix in each tomography solution was considered. Table 7 lists the situation of tomography solution at 12:00 UTC of DOY 182, 2017. We can see that the condition number of coefficient matrix was INF only when the observation equation existed in the tomography model, implying that the matrix has a serious ill-condition. With the addition of horizontal and vertical constraint equations, the condition numbers decreased gradually, indicating that the constraint equations could alleviate the ill-posed issue of the tomography matrix. The condition number increased slightly when the virtual observations of the proposed method were added. The same trend was shown in other tomography solutions due to the existence of multicollinearity between the virtual observation equation and the observation equation. However, the singular value decomposition (SVD) method used in tomography calculation can overcome the problem of unstable results, and the accuracy of the tomography results of the proposed method was still higher than that of the conventional method. Therefore, the method of improving the observation distribution could not optimize the ill-conditioned tomographic matrix, but it caused abundant water vapor information of voxels, which could greatly affect the accuracy of the tomography result. In follow-up research, the ill-condition issue of the coefficient matrix in the tomography equation and the problem of insufficient observations should be considered and distinguished from each other.

## 5. Conclusions

In this paper, an improved GNSS water vapor tomographic method was proposed to improve the distribution of observations. It was validated by tomographic experiments using GNSS data from 12 stations of Hong Kong SatRet for 7 days from DOY 182 to 188, 2017. The influence of the virtual observations on the number of voxels, where signal rays were passing through, aws analyzed. Results showed that the proposed method allowed for more voxels crossed by rays, and the average increase in quantity and proportion was 56 voxels and 10%, respectively. Most of the increased voxels were located in the region of tomographic upper boundary and near the stations.

The comparison between SWV derived from tomographic results and that of GAMIT calculated was conducted using the conventional and proposed method. The MAE, STD, and RMS values decreased from 10.532/11.353/14.087 mm to 8.735/9.696/11.643 mm, respectively. In a comparison of the PWV time series, the MAE, STD, and RMS of the proposed method were smaller than those of the conventional method, whose values were 2.632/2.839/2.937 and 3.839/4.290/4.143 mm, respectively. The water vapor density of the proposed method agreed with that of radiosonde, particularly at the bottom altitudes of the tomographic area, compared with the conventional method. In addition, the results of regression analysis further demonstrated the superiority of the proposed method in obtaining the water vapor density. From the perspective of the ill-conditioned equation, the improvement in observations could not decrease the condition number.

With the effective addition of other observations, including InSAR, water vapor radiometer, and COSMIC radio occultation data, the distribution of observations was further improved. However, whether the condition number of the tomographic matrix was reduced should still be analyzed. Furthermore, the experiments were only carried out in Hong Kong at a specific time period. The feasibility and superiority remain to be further verified for various periods, regions, and weather conditions.

## Figures and Tables

**Table 1:** Information of stations and boundary points.

	Station Name	Latitude	Longitude	Height	Location	Boundary Points of Virtual SWV
**Station for tomographic modeling**	HKKT	22.4449	114.0666	34.5764	Northwest	B, C, D
	HKLT	22.4181	113.9966	125.922		
	HKSL	22.3720	113.9279	95.2972		
	HKPC	22.2849	114.0378	18.1303	Southwest	A, C, D
	HKMW	22.2558	114.0032	194.9461		
	HKNP	22.2491	113.8939	350.6723		
	HKSC	22.3222	114.1412	20.2386	Southeast	A, B, D
	HKOH	22.2477	114.2286	166.4011		
	HKLM	22.2190	114.1201	8.5536		
	HKWS	22.4343	114.3354	63.7909	Northeast	A, B, C
	T430	22.4947	114.1382	41.3228		
	HKST	22.3953	114.1842	258.7045		
**Station for test**	HKSS	22.4311	114.2693	38.7135	Northeast	-
	45004	22.31	114.17	66.0	Southeast	

**Table 2:** GAMIT processing strategy for tropospheric estimation.

Parameter	Strategy
Choice of Observable	LC_AUTCLN
Choice of Experiment	BASELINE
Sampling rate	30 s
Elevation Cutoff	10°
Zenith Model	PWL (piecewise linear)
Tropospheric correction model	Saastamoinen
Mapping Function	GMF
IGS stations	3

**Table 3:** Number and increment of voxels crossed by signal rays in different layers of Solutions #a and #b by two schemes.

	Solution #a			Solution #b		
	Scheme #1	Scheme #2	Increment	Scheme #1	Scheme #2	Increment
1st layer	12	25	10	18	22	4
2nd layer	21	35	14	28	37	9
3rd layer	26	39	13	38	43	5
4th layer	31	40	9	43	46	3
5th layer	34	44	10	42	46	4
6th layer	35	47	12	47	49	2
7th layer	37	46	9	50	52	2
8th layer	41	49	8	52	53	1
9th layer	43	49	6	54	55	1
10th layer	46	49	3	50	56	1

**Table 4:** Accuracies in terms of MAE, STD, and RMS using two schemes for 7 days (Unit: mm).

DOY	MAE		STD		RMS	
	Scheme #1	Scheme #2	Scheme #1	Scheme #2	Scheme #1	Scheme #2
182	11.53	9.66	11.70	9.79	15.75	12.87
183	12.75	9.70	8.47	6.69	14.49	11.17
184	12.37	11.33	15.33	14.87	16.94	15.85
185	10.33	9.57	12.24	11.06	14.05	12.81
186	10.47	9.19	14.68	12.80	15.98	13.32
187	9.73	6.75	9.92	6.82	12.94	9.15
188	6.55	4.95	7.13	5.84	8.46	6.35
Average	10.53	8.74	11.35	9.70	14.09	11.64

**Table 5:** Statistical result of PWV differences between various schemes and radiosonde for 7 days (Unit: mm).

	MAE	STD	RMS
Scheme #1	3.839	4.290	4.143
Scheme #2	2.632	2.839	2.937

**Table 6:** Statistical results of the water vapor density profile comparison between radiosonde and different schemes at UTC 12:00 from DOY 182 to 188, 2017.

DOY	Scheme #1		Scheme #2		Improvement	
	MAE	RMS	MAE	RMS	MAE	RMS
182	1.22	1.34	0.86	1.07	0.36	0.27
183	1.37	1.76	0.89	1.21	0.48	0.55
184	1.81	2.59	0.93	1.79	0.88	0.80
185	1.72	2.46	1.17	1.82	0.45	0.64
186	1.55	2.19	1.04	1.26	0.51	0.93
187	1.54	2.49	1.12	2.21	0.42	0.28
188	1.40	2.01	0.83	1.48	0.57	0.53
**Average**	1.51	2.09	0.98	1.51	0.53	0.57

**Table 7:** Condition number of each coefficient matrix in the tomography solution at 12:00 UTC a.m. DOY 182, 2017.

	$A_{swv}$	$A_{swv}+A_{h}$	$A_{swv}+A_{h}+A_{v}$	$A_{swv}+A_{h}+A_{v}+A_{swv^{\prime }}$
**Condition Number**	INF	2.113 × 10^5^	2.127 × 10^3^	7.055 × 10^3^
